# Outcome of patients with relapsed or refractory acute myeloid leukemia treated with Mito-FLAG salvage chemotherapy

**DOI:** 10.1007/s00432-021-03821-1

**Published:** 2021-10-05

**Authors:** Regina Mühleck, Sebastian Scholl, Inken Hilgendorf, Karin Schrenk, Jakob Hammersen, Jochen J. Frietsch, Maximilian Fleischmann, Herbert G. Sayer, Anita Glaser, Andreas Hochhaus, Ulf Schnetzke

**Affiliations:** 1 Klinik für Innere Medizin II, Abteilung für Hämatologie und Internistische Onkologie, Universitätsklinikum Jena, Am Klinikum 1, 07747 Jena, Germany; 2 4. Medizinische Klinik, HELIOS Klinikum Erfurt, Nordhäuser Straße 74, 99089 Erfurt, Germany; 3 Institut für Humangenetik, Universitätsklinikum Jena, Am Klinikum 1, 07747 Jena, Germany

**Keywords:** AML, Refractory, Relapse, Salvage therapy, Mito-FLAG

## Abstract

**Purpose:**

Curative intended treatment is challenging in patients with relapsed or refractory acute myeloid leukemia (r/r AML) and associated with a dismal prognosis for long-term survival. Despite novel treatment options, the majority of patients are treated with chemotherapy-based regimens. Although widely used, little data exist on the combination of fludarabine, cytarabine, granulocyte colony stimulating factor (FLAG) and mitoxantrone as salvage strategy for r/r AML.

**Materials and methods:**

Sixty-six patients receiving Mito-FLAG for r/r AML treated at a German tertiary care center between 2009 and 2019 were analyzed with regard to response rates, survival and safety profile.

**Results:**

Overall response rate was 75.8% with 56.1% of patients achieving complete remission (CR) and 19.7% partial remission (PR). After a median follow-up of 54 months, median overall survival (OS) was 13 months. Patients transitioned to allogeneic hematopoietic stem cell transplantation (alloHSCT) (75.8%) showed a significant improvement in OS with a median OS of 17 (95% CI 8.5–25.4) months vs 3 (95% CI 1.7–4.3) months (*p* < 0.001). 30- and 60-day mortality rates for all patients after the initial cycle of Mito-FLAG were 4.5% and 7.6%, respectively.

**Conclusion:**

The Mito-FLAG salvage protocol represents an effective and feasible treatment regimen for r/r AML. Importantly, a high rate of transition to successful alloHSCT with the aim of long-term disease-free survival has been shown.

**Supplementary Information:**

The online version contains supplementary material available at 10.1007/s00432-021-03821-1.

## Introduction

Effective salvage therapies present an urgent medical need in patients with relapsed or refractory acute myeloid leukemia (r/r AML) (Döhner et al. 2017; Thol et al. 2015). Intensive induction chemotherapy and consolidation by conventional chemotherapy and/or allogeneic hematopoietic stem cell transplantation (alloHSCT) remain the cornerstone of curative intended AML treatment. 60–80% of younger patients achieve complete remission (CR) following induction chemotherapy. About 50% of patients older than 60 years treated with induction chemotherapy respond adequately (Döhner et al. 2015; Dombret and Gardin 2016).

In contrast, a considerable proportion of patients subsequently relapse and need further treatment. Hence, only about 30–50% of patients younger than 60 years and 10–20% of patients older than 60 years are cured from the disease (Röllig et al. 2020; Short et al. 2018). Recently, new therapeutic agents were implemented into classical “7 plus 3” induction chemotherapy and salvage regimens (Kantarjian et al. 2021; Heuser et al. 2020).

Distinct cytogenetically and molecularly defined entities in AML are crucial in terms of treatment and prognosis of the disease (Döhner et al. 2017; Estey 2020). The heterogenous group of the AML landscape requires different selective therapeutic options.

To date, curative intended r/r AML is treated with intensified (re-) induction protocols, typically comprising high-dose cytarabine as a backbone and different anthracycline and alkylating counterparts (Burnett et al. 2013). A direct comparison of those salvage regimens has not been performed so that a standard regime has not been defined. Commonly applied treatment protocols are fludarabine, cytarabine, granulocyte colony stimulating factor, and idarubicin (FLAG-Ida) and mitoxantrone, etoposide, cytarabine (MEC) (Thol et al. 2015). Rates of achieving CR including CR with incomplete hematologic recovery (CRi) are about 50% (Steinmetz et al. 1999; Westhus et al. 2019; Parker et al. 1997; Spadea et al. 1993). Long-term survival is still dismal with cure rates between 10 and 40% which depend on the option of subsequent alloHCST (Forman and Rowe 2013; Kell 2006; Schmid et al. 2006). A modified FLAG-protocol containing mitoxantrone (Mito-FLAG) has been shown to be effective as a salvage regimen for r/r AML in a multicenter, randomized phase 3 trial comparing cytarabine as bolus versus continuous infusion (Thiel et al. 2015; Hänel et al. 2001). Here, we present efficacy data of Mito-FLAG as a potent chemotherapy protocol even for high-risk patients according to the European LeukemiaNet (ELN) guidelines. In addition, we compare those data with a previous salvage chemotherapy protocol containing high-dose cytarabine and cyclophosphamide (hAC) (Schnetzke et al. 2014).

## Patients and methods

### Patients

Patients with r/r AML (excluding AML M3) who received Mito-FLAG either due to refractory or relapsed disease were analyzed. All patients were treated at University Hospital Jena, Germany, between 2009 and 2019 and included in one of the following AML registries: AML registry of the OSHO study group (East German Study Group of Hematology and Oncology) or in the SAL registry (Study Alliance Leukemia). Patients gave their written consent for data acquisition and analysis after pseudonymization in one of the registries. This retrospective data analysis was approved by the local ethics committee of the University Hospital Jena, Germany (Ethical numbers 4871-07/16 for “retrospective evaluation of therapy response and survival in patients with AML” and 3967-12/13 for SAL registry).

### Mito-FLAG treatment protocol

Mito-FLAG protocol consisted of fludarabine (15 mg/m^2^/bid, 15 min infusion) and cytarabine (1 g/m^2^/bid, 3 h infusion) for 5 consecutive days. In addition, mitoxantrone (7 mg/m^2^, 30 min infusion) was administered on days 1, 3, and 5. Granulocyte colony stimulating factor (G-CSF) was initiated subcutaneously on day 0 until hematologic recovery.

The hAC-regimen was administered as published previously (Schnetzke et al. 2014). Briefly, cytarabine (3 g/m^2^/bid) (3 h infusion) at days 1–4 and cyclophosphamide (1 g/m^2^) on days 1 and 3 were applied.

### Treatment administration

Induction and consolidation chemotherapy were applied either according to the OSHO 2002, OSHO 2004 or “7 + 3” protocol. In younger patients (≤ 60 years), OSHO protocol consisted of idarubicin (12 mg/m^2^, days 1–3) and intermediate-dosed cytarabine (1 g/m^2^/bid, days 1, 3, 5 and 7) as induction chemotherapy, while patients over 60 years received mitoxantrone (10 mg/m^2^, days 1–3) and intermediate-dosed cytarabine (1 g/m^2^/bid, days 1, 3, 5 and 7) as induction treatment. First consolidation chemotherapy in younger AML patients was identical with induction chemotherapy. Elderly AML patients underwent consolidation treatment with a dose reduction of mitoxantrone (10 mg/m^2^, days 1 and 2) and intermediate-dosed cytarabine (0.5 g/m^2^/bid, days 1, 3 and 5) (Büchner et al. 2012; Kahl et al. 2016). Standard “7 + 3” induction regimen was applied as previously published with a continuous 7-day infusion of cytarabine (100 mg/m^2^/day) and daunorubicin (45 mg/m^2^, days 1–3) (Wiernik et al. 1992). For consolidation therapy following “7 + 3”, high-dose cytarabine (3 g/m^2^/bid) for 3 days (day 1, 3, 5) was applied.

AlloHSCT was performed whenever feasible due to the r/r AML as high-risk disease per se.

The majority of patients subsequently undergoing alloHSCT received a reduced-toxicity conditioning (RTC) based on treosulfan or busulfan in combination with fludarabine with or without ATG prior to transplantation (*n* = 42, 84%) (Casper et al. 2012; Kröger et al. 2003). All remaining patients underwent myeloablative conditioning (MAC) (*n* = 8, 16%) (Jethava et al. 2017). The characteristics of patients who underwent alloHSCT and donor type distribution are summarized in Table S1.

### Response evaluation

Overall response rate was defined as complete remission (CR), complete remission with incomplete recovery (CRi) and partial remission (PR). Additional efficacy assessments were performed by calculation for overall survival (OS) and event-free survival (EFS). OS is defined as date of initiation of Mito-FLAG to the date of death from any cause. EFS is the date of initiation of Mito-FLAG to death for any reason, refractory or relapse disease. Response criteria were used according to the ELN 2017 guidelines (Döhner et al. 2017).

Following Mito-FLAG application, first response evaluation was on day 15. Here, a blast count of 5% or less in the bone marrow was assessed as clearance of blasts. Remission evaluation was usually performed after hematologic recovery. CR was defined as 5% blasts or less within the bone marrow and adequate peripheral blood counts (neutrophils ≥ 1.0 × 10^9^/l, platelets ≥ 100 × 10^9^/l), while patients with CRi did not show peripheral hematologic recovery. Partial remission was defined as 5–25% blasts in the bone marrow and a total reduction of blasts of at least 50% of AML blasts. Refractory disease comprises > 25% blasts in the bone marrow or less than 50% reduction defined as persistence of blasts (BP). Failure to attain blast clearance or CR following first-line induction chemotherapy was defined as (primary) refractory disease.

### Safety analyses

Non-hematologic toxicity of Mito-FLAG regimen was evaluated according to the Common Terminology Criteria and Adverse Events (CTCAE v5.0). Evaluation of the hematologic toxicity was performed by analysis of duration of neutrophils below 0.5 × 10^9^/l subsequent to Mito-FLAG application.

### Genetic and molecular analysis

Cytogenetic evaluation was performed with standard banding techniques, and karyotypes were described according to the currently valid International System for Human Cytogenetic Nomenclature (McGowan-Jordan et al. 2021). Cytogenetic categorization into favorable, intermediate or adverse risk was performed on the basis of recommended criteria (Döhner et al. 2017).

The presence of FLT3-ITD and NPM1 mutations was detected by PCR amplification of the corresponding region using genomic DNA followed by fragment analysis (Scholl et al. 2005, 2008).

### Statistical considerations

Binary logistic regression model was conducted to assess the impact of independent variables.

Time-to-event analyses (OS, EFS) were estimated using the Kaplan–Meier method and compared using log-rank test.

Chi^2^-test and Fisher’s exact test were applied for comparing response rates of the Mito-FLAG cohort to the hAC cohort (*n* = 22 patients), whereas survival data were analyzed by log-rank test, respectively. *p* values below 0.05 were considered as statistically significant. Statistical analyses were performed by IBM SPPS v24.0 (SPSS, Chicago, IL, USA).

## Results

### Demographics

Sixty-six patients (female 51.5%, median age at Mito-FLAG initiation 56 years; range 21–71 years) were analyzed for this study (Table 1). All patients retained a good performance status with an ECOG of 2 or less. 34 patients had de novo AML, 23 secondary AML (sAML), and 6 treatment-related AML (tAML). FLT3-ITD diagnostics were performed in 60 patients with 16 of 60 (27%) harboring an activating mutation. Cytogenetics could be carried out in 65 of 66 patients with 6 (9%), 38 (58%) and 21 (32%) considered as low, intermediate and high risk according to cytogenetic risk classification (Table 1).

**Table 1:** Patient demographics

	*n* = 66
Median age at Mito-FLAG, years (range)	56 (21–71)
Sex, female (%)	34 (51.5)
ECOG-performance status, n (%)	
0–1	58 (87.9)
2	5 (7.6)
Unknown	3 (4.5)
AML type, *n* (%)	
De novo	34 (51.5)
sAML	23 (34.9)
tAML	6 (9.1)
Unknown	3 (4.5)
Molecular genetics, *n* (%)	
NPM1 mutation	10 (15.2)
NPM 1 wild type	32 (48.5)
Unknown	24 (36.3)
FLT3-ITD mutation	16 (24.2)
FLT3 wild type	44 (66.7)
Unknown	6 (9.1)
Cytogenetic prognostic group, *n* (%)	
Favorable	6 (9.1)
Intermediate	38 (57.6)
Adverse	21 (31.8)
Unknown	1 (1.5)
ELN-risk group	
Favorable	10 (15.2)
Intermediate	18 (27.3)
Adverse	24 (36.3)
Unknown	14 (21.2)
FAB-classification, *n* (%)	
M0	5 (7.6)
M1/2	30 (45.4)
M4/5	25 (37.9)
M6	1 (1.5)
M7	1 (1.5)
Unknown	4 (6.1)
Induction therapy prior to Mito-FLAG, *n* (%)	
OSHO ≤ 60 years	42 (63.6)
OSHO > 60 years	12 (18.2)
“7 + 3”	10 (15.2)
Other	2 (3)
Consolidation therapy prior to Mito-FLAG, *n* (%)	
None	36 (54.6)
OSHO ≤ 60 years	24 (36.4)
OSHO > 60 years	3 (4.5)
HD-cytarabine	3 (4.5)
Disease status at Mito-FLAG, *n* (%)	
Primary refractory/No CR	36 (54.5)
PR after induction	15 (22.7)
BP after induction	5 (7.6)
No blast clearance @ day 15 of induction	16 (24.2)
Relapse	30 (45.5)
Early relapse	19 (28.8)
Late relapse	11 (16.7)
First relapse	29
Second relapse	1
Relapse after alloHSCT	8

Mito-FLAG: mitoxantrone fludarabine cytarabine granulocyte colony stimulating factor; ECOG: Eastern Cooperative Oncology Group; AML: acute myeloid leukemia; sAML: secondary AML; tAML: therapy-related AML; NPM1: nucleophosmin 1; FLT3-ITD: Fms like tyrosinkinase 3-Internal tandem duplication; ELN: European Leukemia Net; FAB: French–American–British Group; LDH: lactate dehydrogenase; OSHO: Ostdeutsche Studiengruppe Hämatologie und Onkologie; HD-cytarabine: high-dose cytarabine; CR: complete response; PR: partial response; BP: blast persistence; alloHSCT: allogeneic hematopoietic stem cell transplantation; early relapse: relapse within ≤ 12 months; late relapse: relapse after > 12 months CR

### Treatment characteristics and disease status prior to Mito-FLAG

Prior to Mito-FLAG salvage regimen all patients received cytarabine-based induction therapy with 54 (82%) patients according to OSHO protocols and 10 (15%) “7 + 3” regimen. Of the two remaining patients, one received all-trans retinoic acid, idarubicin and cytarabine, whereas the other was treated with CPX-351 as induction therapy.

Prior to Mito-FLAG therapy 30 patients (45%) received at least one cycle of consolidation therapy (Table 1).

Most r/r AML patients were in first salvage with 36 (54.5%) patients who did not achieve CR following induction therapy and 30 (45.5%) patients who received Mito-FLAG due to relapse. Except for one patient, Mito-FLAG was applied for first relapse. Of note, most of relapsing patients presented with early relapse, defined as complete remission for 12 months or less (28.8% early relapse vs 16.7% late relapse) as summarized in Table 1.

### Adverse events

Grade 3 and 4 adverse events (AEs) according to CTCAE occurring in ≥ 10% of patients included febrile neutropenia (86.4%), mucositis (22.7%) and elevated liver enzymes (15.2%) (Table 2). The most common site of infection was pneumonia (48.5%) followed by central line infections.

**Table 2:** Adverse events, *n* (%)

Adverse event	CTCAE-grade 1/2	CTCAE-grade 3/4	CTCAE-grade 5 (death)	Total	ND
Febrile neutropenia/infection	2 (3)	57 (86.4)	4 (6.1)	63 (95.5)	2 (3)
Diarrhea	30 (45.5)	6 (9.1)	0	36 (54.6)	6 (9.1)
Vomiting	27 (40.9)	5 (7.6)	0	32 (48.5)	9 (13.6)
Mucositis	17 (25.8)	15 (22.7)	0	32 (48.5)	9 (13.6)
Cardiac	23 (34.8)	6 (9.1)	0	29 (43.9)	7 (10.6)
Elevation of liver enzymes	40 (60.6)	10 (15.2)	1 (1.5)	51 (77.3)	6 (9.1)
Elevation of creatinine/renal	11 (16.7)	2 (3)	0	13 (19.7)	5 (7.6)
Skin reaction	14 (21.2)	1 (1.5)	0	15 (22.7)	10 (15.2)
Myalgia	3 (4.5)	0	0	3 (4.5)	10 (15.2)
Neurological events	0	4 (6.1)	0	4 (6.1)	9 (13.6)

CTCAE: Common Terminology Criteria for Adverse Events; ND: no data

30-day and 60-day mortality after the initial cycle of Mito-FLAG was 4.5% and 7.6% (3 and 5 patients), respectively. Four patients died of infectious complications, and one patient had a fatal outcome due to liver failure. Subsequent to a second cycle of Mito-FLAG, three more patients died of severe sepsis.

### Efficacy

**Fig. 1:**
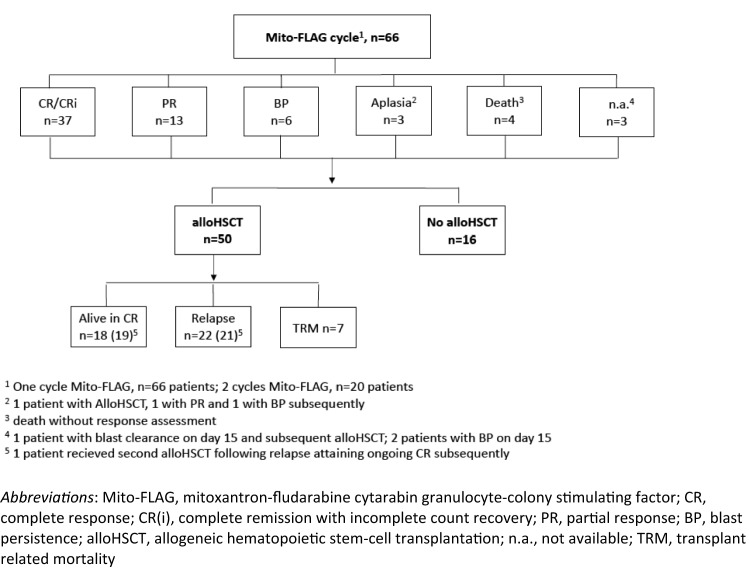
CONSORT diagram, antileukemic responses

**Fig. 2:**
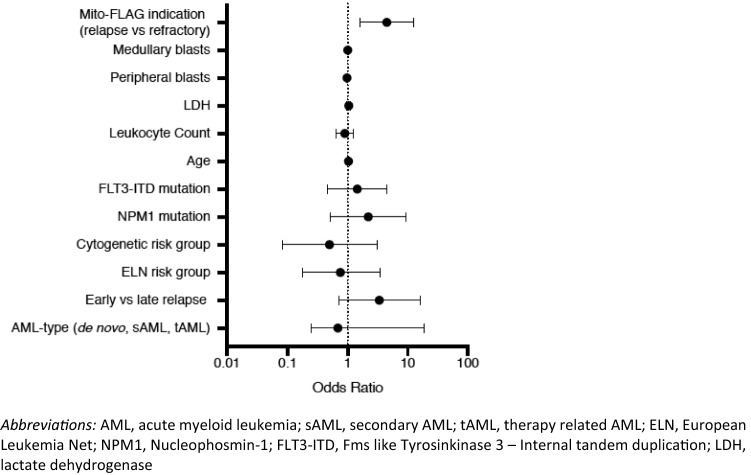
Impact of patient/disease characteristics on achieving complete remission following Mito-FLAG. Impact of different independent variables on achieving complete remission calculated by logistic regression

Figure 1 provides a CONSORT diagram of the Mito-FLAG cohort. CR including CRi (CR with incomplete count recovery) was documented in 37 out of 66 patients (56.1%) and PR in 13 patients (19.7%) (Fig. 1). Six patients (9.1%) demonstrated no reduction in blast count defined as persistence of blasts (BP). Three patients showed an aplastic bone marrow, whereas one out of three underwent alloHSCT and achieved CR subsequently. Several variables were tested by binary logistic regression analysis for correlation achieving CR (Fig. 2) with disease status (refractory or relapse) at the time of Mito-FLAG application being the only variable that was statistically significant. In detail, patients that were treated for refractory disease showed a greater likelihood of achieving CR than relapsed patients (OR 4.49, 95% CI 1.59–12.72, *p* = 0.003). Of note, refractory disease before Mito-FLAG salvage included patients that did not attain CR following induction chemotherapy and patients who did not show blast clearance at day 15 of induction therapy.

**Fig. 3:**
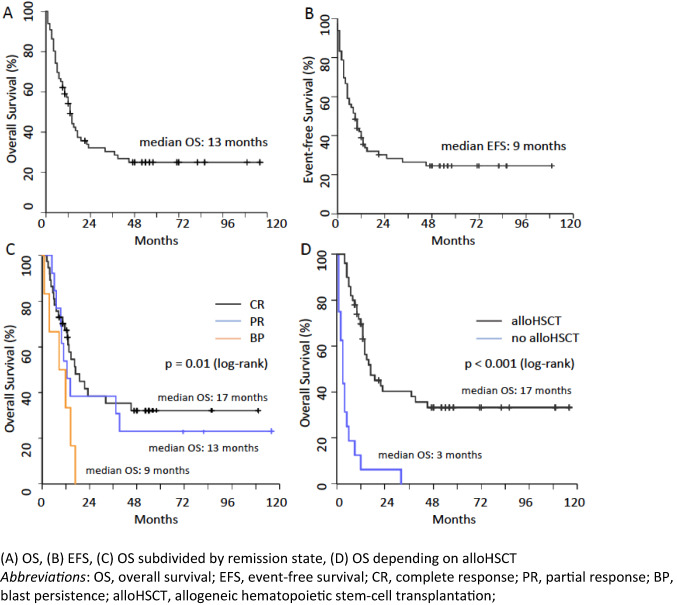
OS and EFS of the whole cohort (**A**–**C**) or alloHSCT patients (**D**) showing months from start of Mito-FLAG

For consolidation, a second Mito-FLAG was applied in 20 out of 66 patients (30.3%). OS and EFS were calculated for the whole Mito-FLAG cohort with a median follow-up of 54 months (Fig. 3). OS at 1, 3 and 5 years was 54.2%, 30% and 25%, respectively. Median OS for the whole cohort of r/r AML patients was 13 (95% CI 10.2–15.8) months (Fig. 3A).

An event (death of any reason, refractory or relapse disease) was noted in 48 patients (72.7%). Median EFS was 9 (95% CI 5.1–12.9) months and probability of 1-, 3- and 5-year EFS was 38.9%, 26% and 24%, respectively (Fig. 3B).

Depending on the response to Mito-FLAG therapy, the median OS was 17 (95% CI 8.6–25.4) months if a CR/CRi, 13 (95% CI 8.3–17.7) months if PR was achieved and 9 (95% CI 0–18.6) months if BP was noted (Fig. 3C). The 1-year probability for OS after a CR/CRi was 70.2%, after PR 53.8% and after BP 33.3%. The 3- and 5-year probability for CR/CRi was 35% and 32.2% and for PR 38.5% and 23.1%. For OS, a significant difference was noted between patients achieving a CR/CRi, PR or BP following Mito-FLAG (*p* = 0.01).

At data cutoff, 19 out of 66 patients (28.8%) were in CR and alive. All of them received an alloHSCT.

### Role of alloHSCT

Following Mito-FLAG, a total of 50 patients (75.8%) transitioned to alloHSCT including 34 patients in CR/CRi (see CONSORT diagram, Fig. 1). Treatment characteristics of alloHSCT patients are demonstrated in Table S1. Of note, 42 of 50 patients (84%) were conditioned by reduced-toxicity/intensity (RTC/RIC) protocols and 8 patients (16%) received myeloablative regimens (MAC). Transplant-related mortality (TRM) was 14% (7 patients) with infectious complications (5 patients) and graft-versus-host-disease (GvHD) (2 patients) as reason of death. At the time of data cutoff 19/50 patients (38%) following alloHSCT were in CR.

Median OS for patients undergoing alloHSCT was 17 (95% CI 8.6–25.4) months (Fig. 3D). Of note, patients transplanted in CR had a median OS of 17 (95% CI 8.03–25.97) months, whereas patients not in CR of 14 (95% CI 0.28–27.72) months (data not shown, n.s.). The probability of surviving 1 year was 75.9% for patients in CR and 56.3% for patients who underwent alloHSCT not in CR (data not shown; n.s.).

Without subsequent alloHSCT, the outcome was significantly worse with a median OS of 3 months (95% CI 1.7–4.3 months, *p* < 0.001). 1-year probability of surviving was 69.7% for patients undergoing alloHSCT and 6.3% for patients who did not undergo alloHSCT. Of note, there was no difference in age between both groups (alloHSCT vs non-alloHSCT).

### Comparison of two different salvage regimens: hAC (high-dose cytarabine and cyclophosphamide) versus Mito-FLAG

A historical cohort of 22 patients who received high-dose cytarabine (3 g/m^2^, q12h, days 1–4) and cyclophosphamide (1 g/m^2^) days 1 and 3 between 2000 and 2013 was compared to 62 patients of the Mito-FLAG cohort regarding efficacy. Patients who received both protocols were not considered (one patient of the hAC- and 4 patients of the Mito-FLAG cohort).

**Fig. 4:**
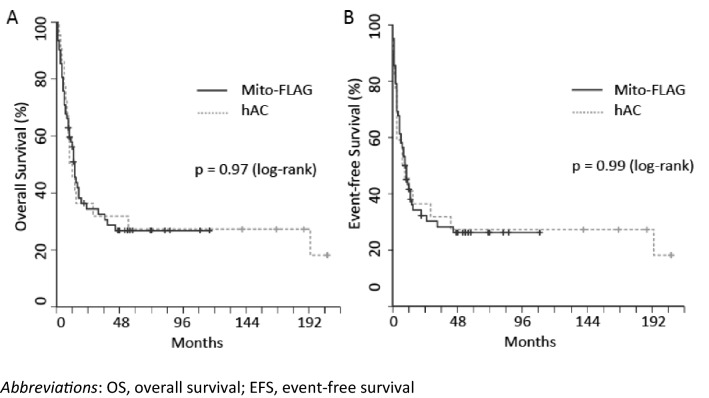
OS (**A**) and EFS (**B**) of Mito-FLAG and hAC cohort

Patient characteristics for both groups are presented in Table S2. Importantly, the median age was 57 years for the Mito-FLAG group and 40 years for the hAC group. Indication for salvage therapy was comparable with 50% (11 patients) refractory and 50% relapsed AML patients in the hAC group. Overall response rates were comparable for achieving CR/CRi and PR (Table S3). Interestingly, OS and EFS were comparable with respect to different treatment cohorts (Fig. 4A, B).

## Discussion

Despite achieving high CR rates by induction chemotherapy prognosis of r/r AML remains unsatisfying (Thiel et al. 2015; Burnett et al. 2015; Fernandez et al. 2009; Megías-Vericat et al. 2018). Curative intended treatment for r/r AML consists of salvage chemotherapy strategies, typically containing a high-dose cytarabine backbone, e.g., FLAG-Ida or MEC regimen (Döhner et al. 2017; Thol et al. 2015). For distinct subgroups, targeted agents have been approved such as gilteritinib in FLT3-mutated patients (Perl et al. 2019). A direct comparison between conventional intensive chemotherapy-based protocols has not been performed. Therefore, salvage regimens mainly depend on physician’s choice and patient’s characteristics. Subsequently, alloHSCT has been identified to be the only option of consolidation treatment with the potential to cure r/r AML (Thol et al. 2015).

The Mito-FLAG protocol represents a standard salvage chemotherapy regimen for r/r AML, but only little data on efficacy exist (Thiel et al. 2015). Here, a detailed analysis of 66 adult r/r AML patients treated at a German tertiary care center receiving Mito-FLAG is presented with regard to safety and outcome. A particular focus was set on patients undergoing alloHSCT. Without this type of consolidation treatment, remission following salvage regimens happens to be only short term (Heuser et al. 2020; Megías-Vericat et al. 2018).

Here, Mito-FLAG salvage treatment resulted in an ORR of 75.8% including 56.1% patients in CR/CRi and 19.7% in PR, respectively. A multicenter, randomized phase III trial comparing cytarabine as bolus versus continuous infusion in r/r AML patients represents the most extensive work on Mito-FLAG to date (Heuser et al. 2020; Megías-Vericat et al. 2018). Of 252 patients, 128 were treated with cytarabine bolus application, and a CR rate of 54% and PR of 15.7%, respectively, was documented which is in accordance to ORR of the Mito-FLAG analysis presented here. A second study published on Mito-FLAG comprised 45 patients with 47% achieving CR following Mito-FLAG (Luo et al. 2013). Of note, refractory patients were not included in that study and no information on genetics or type of AML was provided.

In general, responses in r/r AML vary greatly (CR rates from 20 to 60%) by treatment selection, line of therapy and patient characteristics (Döhner et al. 2017; Roboz et al. 2014). A meta-analysis exploring different salvage regimens did reveal CR rates of 44–66% (Megías-Vericat et al. 2018). The combination of high-dose cytarabine and cyclophosphamide (hAC) was shown to be another potent salvage protocol (Schnetzke et al. 2014).

For this study we compared the response rates and outcome of 22 hAC patients and 62 Mito-FLAG patients who were all treated at the same center. Patients who received both protocols due to refractoriness to one or the other were excluded. This resulted in the exclusion of one patient of the hAC- and four patients of the Mito-FLAG cohort, respectively. Furthermore, patients who received iAC (intermediate-dose cytarabine and cylcophosphamide) were also excluded since cytarabine doses of iAC are not comparable to cytarabine doses used within the Mito-FLAG regimen. With regard to the limitations of comparing those two cohorts, no statistically significant differences were seen between both salvage regimes (CR rates 54.8% Mito-FLAG and 54.5% hAC, respectively).

Refractory versus relapsed AML was the only parameter carrying an impact of achieving remission at the time of Mito-FLAG therapy. While refractory disease was associated with higher probability of CR, this might be in part explained by the fact that 8 of 30 patients who were treated for relapsed AML already underwent alloHSCT prior to Mito-FLAG which harbors a worse prognosis per se. Also, 15 of 36 patients who were treated due to “primary refractory” disease showed in fact PR. Although the definition of refractory disease is not well defined and differs in clinical practice and trials, failure to attain CR to intensive induction therapy is recognized as “primary refractory” (Döhner et al. 2017). A third explanation is that patients not showing complete blast clearance at day 15 bone marrow examination post-induction chemotherapy were considered as not optimal responding and received Mito-FLAG. Out of 16 patients who belong in this category, several patients had shown blast reduction without complete clearance and therefore were treated with Mito-FLAG salvage regimen.

The 30-day and 60-day mortality was 4.5% (*n* = 3) and 7.6% (*n* = 5) following first cycle of Mito-FLAG with four patients dying of infectious complications and another patient died of liver failure.

Febrile neutropenia, infectious complications, mucositis and moderate increase in liver enzymes accounted for the majority of adverse events. When comparing with a large analysis of FLAG-Ida salvage therapy, 30- and 60-day mortality was 9.1% and 15.9%, respectively (Westhus et al. 2019). Owing to the improvements in supportive care especially antimicrobial prophylaxis Mito-FLAG salvage regimen is a feasible and well-tolerated chemotherapy protocol.

With a median follow-up of 54 months, survival analyses demonstrated a median OS of 13 months with a 1-year, 3-year and 5-year OS of 54.2%, 30% and 25%, respectively. Importantly, 75.8% (*n* = 50) patients subsequently underwent alloHSCT. The importance of alloHSCT as an essential cornerstone for curative intended treatment is underlined by the fact that all patients that are alive at data cutoff (*n* = 19) were transplanted.

Of note, by log-rank-test no statistical difference was seen when survival analyses of the Mito-FLAG cohort were compared to the hAC cohort treated at the same center. Mito-FLAG patients were notably older than hAC patients. Considering statistical limitations by comparing those two cohorts, it should be pointed out that both salvage regimens represent well tolerated and efficacious treatment options for r/r AML.

To date, new treatment strategies for distinct molecular subgroups are available. Gilteritinib, an FLT3-inhibitor led to higher percentages of patients with responses and longer survival than salvage chemotherapy among patients with r/r FLT3-mutated AML. In a non-randomized trial the addition of the Bcl-2 inhibitor venetoclax to the FLAG-Ida regimen improved historical outcomes of FLAG-Ida alone, especially when applied as first salvage (DiNardo et al. 2021).

In conclusion, Mito-FLAG therapy is a well-tolerated salvage regimen leading to high response rates in r/r AML. AlloHSCT remains essential as consolidation treatment for long-term disease-free survival in r/r AML patients.

## Supplementary Information

Below is the link to the electronic supplementary material.

Supplementary file1 (DOCX 26 KB)

## Data Availability

Date are available from the patient records of University Hospital Jena.
