# Supplementary material for: Outcome of patients with relapsed or refractory acute myeloid leukemia treated with Mito-FLAG salvage chemotherapy
Source: J Cancer Res Clin Oncol. 2021 Oct 5;148(9):2539–48. doi: 10.1007/s00432-021-03821-1 (PMC9349069; doi:10.1007/s00432-021-03821-1)
Supplement: Supplementary file 1 — Supplementary file1 (DOCX 26 KB) [file 432_2021_3821_MOESM1_ESM.docx]

**Table S1: Characteristics of Mito-FLAG patients subsequently undergoing alloHSCT**

| Parameter |  | n=50 |
| --- | --- | --- |
| Age at alloHSCT, Median (range) n (%) |  | 56 (22-72) |
| Remission prior alloHSCT, n (%) | 1st CR/ CRi 2nd CR/CRi PR BP Aplasia | 23 (46)  11 (22)  10 (20)  4 (8)  2 (4) |
| Donor source, n (%) | MRD MUD  mMUD  haplo-alloHSCT | 7 (14)  27 (54)  15 (30) 1 (2) |
| Conditioning intensity, n (%) | RTC  MAC | 42 (84) 8 (16) |
| HCT-CI, (Sorror et al. 2005), n (%) | 0  1-2 ≥ 3 | 14 (28)  23 (46)  13 (26) |
| Severity of GvHD, n (%) | acute GvHD no or grade I  grade II-IV | 35 (70)  15 (30) |
|  | chronic GvHD mild moderate severe | 4 (8) 5 (10) 6 (12) |
| Mortality, n (%) | **TRM**  yes  no | 7 (14) 43 (86) |
|  | **NRM** yes no  ND | 9 (18)  40 (80) 1 (2) |

*Abbreviations*: alloHSCT, allogeneic stem-cell transplantation; CR(i), complete remission (incomplete count recovery); PR, partial remission; BP, blast persistence; MRD, matched related donor; MUD, matched unrelated donor; mMUD, mismatched unrelated donor; haplo-alloHSCT, haploidentical alloHSCT; RTC, reduced-toxicity conditioning; MAC, myeloablative conditioning; HCT-CI, Hematopoietic Cell Transplantation-specific Comorbidity Index; GvHD, Graft-versus-Host-Disease; TRM, transplant-related mortality; NRM non-relapse mortality; ND, no data

**Table S2: Characteristics of both Mito-FLAG cohort and hAC cohort.**

| Parameter |  | Mito-FLAG-cohort  n=62 | hAC-cohort  n=22 |
| --- | --- | --- | --- |
| Age, years median (range) | at salvage therapy | 57 (21-71) | 40 (21-56) |
| sex, n (%) | male  female | 31 (50) 31 (50) | 11 (50) 11 (50) |
| AML-type, n (%) | *de novo*  sAML  tAML unknown | 32 (51.6)  22 (35.5)  6 (9.7)  2 (3.2) | 19 (86.4) 3 (13.6) 0 (0)  0 (0) |
| Cytogenetic risk group, n (%) | favorable  intermediate  adverse  ND | 6 (9.7) 35 (56.4) 20 (32.3) 1 (1.6) | 2 (9.1) 13 (59.1) 6 (27.3) 1 (4.5) |
| Molecular genetics, n (%) | **FLT3-ITD** mutated  wildtype unknown | 16 (25.8) 41 (66.1) 5 (8.1) | 6 (27.3) 13 (59.1) 3 (13.6) |
| FAB-classification | M0 | 4 (6.5) | 4 (18.2) |
|  | M1/2 | 28 (45.2) | 11 (50) |
|  | M3 | 0 (0) | 1 (4.5) |
|  | M4/5 | 25 (40.2) | 6 (27.3) |
|  | M6 | 1 (1.6) | 0 (0) |
|  | M7 | 1 (1.6) | 0 (0) |
|  | unknown | 3 (4.8) | 0 (0) |

*Abbreviations*: hAC, high dose cytarabin cylophosphamid; Mito-FLAG, mitoxantron-fludarabine cytarabin granulocyte-colony stimulating factor; AML, acute myeloid leukemia; sAML, secondary AML; tAML, therapy related AML; FLT3-ITD, Fms like Tyrosinkinase 3 – Internal tandem duplication; ND, no data; FAB, French-American-British classification

**Table S3: Response rates Mito-FLAG vs hAC cohort**

| Response | Mito-FLAG-cohort  n=62 | hAC-cohort  n=22 | *P* value |
| --- | --- | --- | --- |
| CR/CRi, n (%) | 34 (54.8) | 12 (54.5) | .981 |
| PR, n (%) | 13 (21) | 4 (18.2) | 1.0 |
| BP, n (%) | 5 (8.1) | 6 (27.3) | **.032** |
| Death, n (%) | 4 (6.5) | 0 (0) |  |
| Aplasia, n (%) | 3 (4.8) | 0 (0) |  |
| ND | 3 (4.8) | 0 (0) |  |

*Abbreviations*: CR(i), complete remission (incomplete count recovery); PR, partial remission; BP, blast persistence; ND, no data
